# Thrombotic events and COVID-19 vaccines

**DOI:** 10.5588/ijtld.21.0298

**Published:** 2021-09-01

**Authors:** C. Brazete, A. Aguiar, I. Furtado, R. Duarte

**Affiliations:** 1 Instituto de Saúde Pública da Universidade do Porto, Porto, Portugal; 2 Unidade de Saúde Pública do Alto Minho, Viana do Castelo, Portugal; 3 EPIUnit, Instituto de Saúde Pública da Universidade do Porto, Porto, Portugal; 4 Laboratório para a Investigação Integrativa e Translacional em Saúde Populacional (ITR), Porto, Portugal; 5 Serviço de Infeciologia, Centro Hospitalar e Universitário do Porto, Porto, Portugal; 6 Unidade de Investigação Clínica da Administração Regional de Saúde do Norte, Porto, Portugal; 7 Departamento de Ciências da Saúde Pública, Ciências Forenses e Educação Médica, Universidade do Porto, Porto, Portugal; 8 Serviço de Pneumologia, Centro Hospitalar de Vila Nova de Gaia/Espinho, Vila Nova de Gaia, Portugal

**Keywords:** COVID-19, SARS-CoV-2, vaccines, thrombosis, review

## Abstract

COVID-19 vaccines are considered promising agents in the control of the pandemic. Although their safety was assessed in randomised clinical trials, severe adverse events (AEs) have been reported after large-scale administration. This study aims to evaluate thromboembolic AEs reported after vaccination in a real-world context and how they led to the interruption of vaccination campaigns. We also review the benefits and risks of the vaccines approved in the European Union and provide recommendations. A review of the literature was performed using Medline/PubMed electronic database as well as institutional and pharmaco-vigilance official reports. Our findings show that vaccine-induced prothrombotic immune thrombocytopenia has been suggested as a very rare AE associated with viral vector vaccines. Unusual thrombotic events combined with moderate-to-severe thrombocytopenia were reported mainly in women under 60 years of age. As safety signals emerged, Vaxzevria and Janssen’s COVID-19 vaccine campaigns have been paused while investigations proceed. On the other hand, the number of deep vein thrombosis and pulmonary embolism reports have not increased. Post-marketing surveillance indicated that mRNA vaccines are safe and should continue to be used. The thrombotic events report rate is not increased in people over 60 years. As they are at greater risk for COVID-19 complications and death, no vaccine restrictions are recommended in this group. Risk factors for vaccine-induced prothrombotic immune thrombocytopenia should be established so that evidence-based decisions can be made. Systematic monitoring of COVID-19 vaccine safety is essential to ensure that the benefits of vaccination outweigh the risks.

## Abstract (FR)

Les vaccins contre la COVID-19 sont considérés comme des agents prometteurs du contrôle de la pandémie. Bien que leur innocuité ait été évaluée dans des essais cliniques randomisés, des évènements indésirables (AE) graves ont été rapportés après l’administration de ces vaccins à grande échelle. Cette étude a pour objectif d’évaluer les AE thromboemboliques rapportés après vaccination en vie réelle et de comprendre comment ces AE ont entraîné l’arrêt des campagnes de vaccination. Nous examinons également les bénéfices et les risques des vaccins homologués dans l’Union européenne et fournissons des recommandations. Une revue de la littérature a été réalisée en utilisant la base de données électronique Medline/PubMed, ainsi que les rapports officiels institutionnels et de pharmacovigilance. Nos résultats montrent que la thrombocytopénie immunitaire prothrombotique induite par les vaccins a été suggérée comme AE très rare associé aux vaccins à vecteur viral. Des évènements thrombotiques atypiques associés à une thrombocytopénie modérée à sévère ont été rapportés, principalement chez des femmes de moins de 60 ans. À mesure que des signaux de sécurité étaient émis, les campagnes de vaccination contre la COVID-19 avec les vaccins Vaxzevria et Janssen ont été interrompues le temps des investigations. Toutefois, le nombre de rapports de thrombose veineuse profonde et d’embolie pulmonaire n’a pas augmenté. Les rapports de surveillance post-mise sur le marché indiquaient que les vaccins à ARN messager étaient sans danger et qu’ils devaient continuer à être administrés. Le taux de rapport d’évènements thrombotiques n’est pas plus élevé chez les personnes âgées de plus de 60 ans. Puisque ces dernières sont à risque accru de complications et de décès en cas de COVID-19, aucune restriction vaccinale n’est recommandée dans ce groupe populationnel. Les facteurs de risque de thrombocytopénie immunitaire prothrombotique induite par les vaccins devraient être définis afin de pouvoir prendre des décisions factuelles. Le suivi systématique de l’innocuité des vaccins contre la COVID-19 est essentiel afin de garantir que les bénéfices de la vaccination l’emportent sur les risques.

Severe acute respiratory syndrome coronavirus 2 (SARS-CoV-2) has caused an unprecedented pandemic, severely impacting public health and the economy worldwide. According to data from the World Health Organization, as of 9 May 2021, there were 157,289,118 confirmed cases of coronavirus disease 2019 (COVID-19) and 3,277,272 deaths.^1^ Although the disease was recognised for over a year, no effective treatment or cure is available yet. Therefore, prevention is the cornerstone of the fight against COVID-19. Currently, there are about 200 vaccines under development worldwide; 91 of them are in clinical trial, and four have already been approved by the European Medicines Agency (EMA; Amsterdam, The Netherlands).^2^ While this rapid development of the COVID-19 vaccines is an encouraging step towards the end of the pandemic, it has raised several safety concerns.

Developed by BioNTech (Mainz, Germany) and Pfizer (New Brunswick, NJ, USA), Comirnaty (BNT162b1) was the first COVID-19 vaccine granted conditional marketing authorisation worldwide. Approved on 21 December 2020 by the EMA for people aged ≥16 years, it rapidly became available to most State Members. Clinical trials reported an effectiveness of 95% and a favourable safety profile, with adverse events (AEs) in less than 30% of the vaccine recipients, most of them being mild and transient.^3^ On 6 January 2021, the European Commission authorised the second vaccine against COVID-19, the Moderna (Cambridge, MA, USA) mRNA-1273, for people aged ≥18 years. The Moderna vaccine had an effectiveness of 94.5% in preventing SARS-CoV-2 infection.^4^ BNT162b1 and mRNA-1273 were the first commercialised vaccines in history to use messenger RNA technology.^5^ Their safety and effectiveness were demonstrated in Phase III randomised clinical trials.^3^,^4^

On 29 January 2021, the ChAdOx1 nCov-19 vaccine from AstraZeneca (Cambridge, UK) was the third COVID-19 vaccine to be approved in Europe. The company recently announced the results of Phase III clinical trial, which found the vaccine to be 76% effective.^6^,^7^ The most recently approved vaccine in Europe is the Ad26.COV2-S, developed by Janssen Pharmaceutica (Beerse, Belgium), one of the Janssen Pharmaceutical Companies of Johnson & Johnson (New Brunswick, NJ, USA), with an efficacy of 66% in trials.^8^ Both AstraZeneca and Janssen produced viral-based vector vaccines approved for people aged ≥18 years.^9^ The Figure shows the milestones in the implementation of the approved vaccines in Europe.

**Figure:**
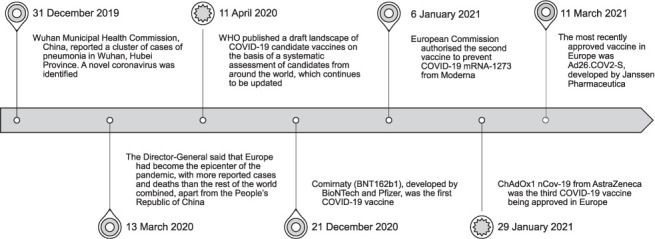
Timeline of the implementation of the vaccines in Europe.

Overall, the AEs found on COVID-19 vaccine trials were mainly mild to moderate and transient, and resolved within a few days after vaccination. The most frequent local adverse reactions were pain and tenderness at the injection site. Common systemic side effects included fatigue, fever, myalgias and headache.^9^,^10^,^11^ Although these vaccines were considered safe in trials, post-marketing surveillance has an important role in identifying rare AEs, which may be clinically relevant. EudraVigilance is a passive surveillance system created by the EMA that collects reports on the AEs of authorised medicines in Europe. Competent national authorities and marketing authorisation holders may report suspected serious adverse reactions occurring within and outside the European Economic Area (EEA). The information is publicly available on its website.^12^ There is an analogous system in the United States specific for vaccines – the Vaccine Adverse Event Reporting System (VAERS). While vaccine safety in real-world contexts has already been reviewed elsewhere,^13^,^14^ safety signals regarding thromboembolic events have emerged more recently, leading to the interruption of some vaccination campaigns.

The present study aimed to review the available scientific information about the benefits and risks of the four COVID-19 vaccines approved in Europe, particularly the thromboembolic AEs reported after their rollout and the subsequent governmental and institutional procedures.

## MATERIALS AND METHODS

A comprehensive literature search was performed using Medline/PubMed electronic database to identify articles describing thromboembolic events after vaccination against SARS-CoV-2. The articles published from 2020 up to 30 April 2021 were included. The terms searched were “covid-19”, “sars-cov-2”, “vaccine”, “vaccination”, “adverse effects”, “side effects”, “thrombosis”, “thrombotic”, “embolism”, “embolic” and “thromboembolism” in “Title/abstract” section. Ethics approval was not required for this study as this was non-human research. The results were narratively summarised.

## RESULTS

There were 252 relevant articles published until 30 April 2021. After screening, 18 papers were included. Relevant studies found by backward citation tracking were also included. Safety reports from the EMA and the US Food and Drug Administration (FDA) were also reviewed. Original data were collected from the European pharmacovigilance database, EudraVigilance. Table 1 describes the general characteristics of the COVID-19 vaccines approved in Europe.

**Table 1:** Overview of general characteristics of the COVID-19 vaccines approved in Europe

	Comirnaty	Moderna	Vaxzevria	Janssen^*^
Type	mRNA	mRNA	Viral vector	Viral vector
Age, years	≥16	≥18	≥18	≥18
Number of doses	2	2	2	1
Efficacy, %	95	94.5	76	66
Storage	−90°C to −60°C	−25°C to −15°C	2°C to 8°C	−25°C to −15°C^*^
Price per dose, US$	14.70–19.90	15–18	2.15–5.25	8.50

^*^ COVID-19 vaccine Janssen can be stored at 2°C to 8°C for 3 months. Sources:^15^,^39^

### Vaxzevria

AstraZeneca and the University of Oxford produced Vaxzevria, a low-cost vaccine distributed on a large scale, including low-middle-income countries. Contrary to Comirnaty, it does not require extremely low temperatures for storage, as it can be stored in an ordinary refrigerator.^6^,^15^ However, despite these benefits, it has recently been implicated in controversy. From 11 March 2021, several countries temporarily suspended the vaccine due to reports of severe coagulation disorders after administration, some of which resulted in death.^16^,^17^ In the meantime, three case series were published reporting unusual thrombotic events combined with thrombocytopenia diagnosed in Germany, Austria, Norway and the United Kingdom after vaccination with ChAdOx1 nCoV-19.^18^–^20^ Patients developed one or more thrombotic events, either venous or arterial, mainly cerebral venous sinus thrombosis (CVST), splanchnic vein thrombosis, pulmonary embolism, deep vein thrombosis (DVT) and ischaemic stroke. CVST was the most common thrombotic event reported, and was often followed by secondary cerebral haemorrhage and disseminated intravascular coagulation. Blood tests showed low platelet counts, very high D-dimer levels, superior to what is found in DVT, and low to normal fibrinogen levels. Confirmatory enzyme-linked immunosorbent assay tests showed an increase in antibodies against platelet factor 4 (PF4), which presumably activated a large number of platelets and caused a rare form of immune thrombotic thrombocytopenia, later named vaccine-induced prothrombotic immune thrombocytopenia (VIPIT). This constellation of thrombotic disorders concurrently with thrombocytopenia and antibodies against PF4 resembles atypical or autoimmune heparin-induced thrombocytopenia, which happens even in the absence of previous exposure to heparin.^21^

Furthermore, other case reports have been published. A 50-year-old male presented in an Italian hospital with a severe headache 7 days after the first dose of the vaccine. Computed tomography (CT) scan revealed cerebral haemorrhage and CVST. The patient underwent urgent neurosurgical intervention. However, he died 18 h later.^22^ In the United Kingdom, two males aged 25 and 32 years developed severe thrombocytopenia and fatal CVST following vacci-nation.^23^ A 60-year-old woman was admitted to a Danish hospital with severe persistent abdominal pain 7 days after the first dose of Vaxzevria.^24^ ACT scan showed bilateral adrenal haemorrhages and a subcapsular and renal haematoma. In the following days, she developed infarction of the area supplied by the right middle cerebral artery. Laboratory findings were compatible with VIPIT, and despite the intensive care treatment, she died on Day 6 after hospitalisation. A woman in her thirties developed a headache 1 week after vaccination with ChAdOx1 nCov-19.^25^ Her condition deteriorated rapidly, and she presented in the emergency department with reduced consciousness, speech impairment and uncoordinated movements. A CT scan of the head showed extensive right-sided haemorrhage and incipient herniation, which lead to her death the next day. Laboratory results were compatible with a diagnosis of VIPIT. Postmortem examination revealed fresh small thrombi in the transverse sinus, frontal lobe and pulmonary artery.

The 45 cases included in this review occurred within 4–26 days after the first dose of Vaxzevria. Most of the patients were women under 60 years of age, a third of whom had predisposing factors for thrombosis, such as hormonal contraception or substitution therapy, Hashimoto thyroiditis, hypertension and recent pregnancy. Other prothrombotic comorbidities identified were Von Willebrand disease and primary sclerosing cholangitis. Although most of the patients were previously healthy, the case-fatality rate of the 45 cases included in this review was 44%.

The AstraZeneca vaccine was later renamed Vaxzevria. The European Pharmacovigilance Risk Assessment Committee has evaluated this safety signal and concluded that a causal association between vaccination with Vaxzevria and very rare cases of thrombosis is plausible.^26^ It recommended an update of the product information for Vaxzevria to specify thrombocytopenia as a new common side effect (occurring in less than 1 in 10 persons) and thrombosis in combination with thrombocytopenia as a new, very rare side effect (occurring in less than 1 in 10,000 persons). VIPIT mainly occurred in women under 55 years of age; however, no other risk factors have been identified.^26^,^27^ Therefore, many countries, such as Germany, Italy, Belgium, Spain and Portugal, restricted the vaccine to people aged 50–60 years and over. Table 2 shows the safety of COVID-19 vaccines approved in Europe.

**Table 2:** Safety of COVID-19 vaccines approved in Europe

	Comirnaty	Moderna	Vaxzvria	Janssen
AEs clinical trials, %				
All	27	23.9	NA^*^	17–35
Severe	<4	<20	2.1	1.7
Serious	0.6	0.6	0.9	0.6
AEs real-world data, *n*				
Mild/moderate (A)	105,686	9,900	85,287	595
Severe (B)	56,209	10,227	115,294	989
Ratio (B/A), %	53	103	135	166
Most frequent local side-effect	Pain at injection site	Pain at injection site	Pain at injection site and tenderness	Pain at injection site
Most frequent systemic side-effects	Fatigue and headache	Fatigue and headache	Fatigue, headache, feverishness, and myalgia	Fatigue, headache, and myalgia
Total number of AEs reported to EudraVigilance, *n*^†^	161,895	20,127	200,581	1,575
Number of CVST	48	4	260	11
Number of DVT	517	96	1,012	44
Number of pulmonary embolisms	866	231	1,281	68

^*^ This study could not obtain adverse reaction rate.^3^,^4^,^8^,^11^

^†^ Data from EudraVigilance database as of 1 May 2021.

AE = adverse event; NA = not available; CVST =cerebral venous sinus thrombosis; DVT = deep vein thrombosis.

Among 34 million vaccines administered in European Union/European Economic Area and the United Kingdom from 11 February to 4 April, several thrombotic events have been reported to EudraVigilance, including 169 CVST.^27^ According to recent studies, the incidence of this condition is 15 cases per million people per year.^28^ Therefore, 5 cases per million people could be expected in 4 months. VIPIT is estimated to occur in 1 in every 100,000 vaccinated people.^21^,^29^ However, the risk is higher in younger people and doubles for people aged between 40 and 49 years.^29^ Thus, there have been more CVST cases after vaccination with Vaxzevria than would be expected for this rare type of thrombosis.

However, more common thrombotic events do not seem to be overrepresented in vaccinated people. It has been suggested that the incidence of pulmonary embolism and DVT in patients vaccinated with ChAdOx1 nCov-19 is not increased compared to the general population.^30^ A multinational cohort study (currently under peer review) evaluated the AEs reported in electronic medical records after anti-COVID-19 vaccination in a real-world context. DVT incidence rates were consistently under 1/100 to 1/10,000, except for people aged ≥85 years. Pulmonary embolism incidence rate also increased with age, but remained under 1/100 to 1/10,000 among all age groups.^12^

EMA’s safety committee analysed the risks of thrombotic complications by age group in the context of high, medium and low infection rates compared to the benefits in terms of hospitalisations due to COVID-19, intensive care unit (ICU) admissions and deaths. Their assessment indicated that the benefits increase with increasing age and higher incidence rates. The committee considered that the benefits of vaccination outweigh the risks. Nevertheless, they continue to analyse new data from the surveillance systems.^27^,^29^ On the other hand, the US FDA has not authorised this vaccine yet.

### Janssen’s COVID-19 vaccine

Johnson & Johnson (Janssen) COVID-19 vaccine was authorised in the United States in late February, and then in Europe in mid-March. Clinical trials placed in different countries showed an efficacy of 66% in preventing symptomatic COVID-19 with a single dose, which represents an advantage over the previously approved vaccines.^8^,^31^ Furthermore, it costs the EU only US$8.50 a dose, approximately four times cheaper than the first two approved vaccines, which are double-dose, costing US$14–US$18 each.^15^ Nonetheless, its efficacy is lower compared to the Pfizer and Moderna vaccines. Moreover, on 13 April 2021, the Centers for Disease Control and Prevention (CDC), together with the FDA, recommended a pause in the use of the Janssen vaccine as a precaution after six reported cases of “a rare and severe type of blood clot in people who received the vaccine”.^32^ An investigation started and, on 20 April, the EMA reported a possible link between vaccination with the Janssen vaccine and unusual thrombotic events, combined with thrombocytopenia.^33^ Indeed, these reactions were similar to those described previously after vaccination with Vaxzevria, including CSVT and splanchnic veins thrombosis (Table 2). Among 7 million people inoculated with Janssen’s vaccine in the United States, eight severe thrombotic events were reported, one of which was fatal. All cases occurred in people under 60 years and, mostly in women. This would suggest that the CVST reporting rate was lower than for the general population. However, among 1,402,712 doses administered to women aged 20–50 years in the United States, observed cases of CSVT exceed by three-fold or greater than the expected cases.^34^ The EMA’s safety committee concluded that the product information should be updated, including these events as very rare side effects.^33^ Health professionals should be aware of them so that VIPIT can be diagnosed and promptly treated. Consultation with haematology specialists is strongly recommended. The treatment algorithms proposed include four axes – administering high-dose intravenous immune globulin or plasma exchange to reduce the pathological antibodies levels; avoiding platelet transfusions; avoiding the administration of heparin as a precaution, and using direct oral anti-Xa inhibitors as preferred first-line anticoagulants.^20^,^35^,^36^ On 23 April, the FDA lifted the suspension of the Janssen COVID-19 vaccine and amended the product information to include information about “a very rare and serious type of blood clot in people who receive the vaccine”.^37^ Given the risk of hospitalisation and death from COVID-19 and the rarity of these AEs, the EMA still recommends this vaccine, as the benefits outweigh the risks.

### Comirnaty

Polack described Phase II/III largest cohort results evaluating a COVID-19 vaccine to that date, with over 43,000 patients.^3^ To date, Comirnaty has the highest effectiveness among the approved vaccines. However, it is more expensive than the more recently authorised ones, requires two doses and must be stored at –60°C, creating challenges for reliable cold chain distribution.^38^,^39^ The first case of DVT after vaccination with Comirnaty was reported in February.^40^ Other thrombotic cases have later been reported for both mRNA vaccines. The EMA is closely monitoring these side effects. However, their incidence is lower than would be expected, and clinical features are different from VIPIT. Therefore, the current evidence does not suggest a causal relationship.^41^ A case of immune thrombocytopenia in a 22-year-old after vaccination with the Pfizer/BioNTech mRNA vaccine was reported in January 2021.^42^ Presentation symptoms included widespread petechiae and gum bleeding. Severe thrombocytopenia was the most relevant laboratory finding. The patient was discharged on Day 3 after treatment and was healthy without autoimmune disease at follow-up. Later, other cases of immune thrombocytopenia after exposure to vaccines from Pfizer-BioNTech and Moderna have been reported,^43^ and a safety signal emerged. Contrary to VIPIT, this case was not accompanied by thrombotic events. Although temporal relationship suggests that the vaccine may have caused these AEs, it could also be coincidental as the incidence is about 3,3 per 100,000 adults/year.^44^

### Moderna COVID-19 vaccine

Similarly to Comirnaty, the vaccine from Moderna demonstrated high effectiveness after two doses, although at a higher price point, compared to the viral vector vaccines.^15^ An observational study using data from passive and active surveillance systems reported that after the first month of vaccination with Pfizer/BioNTech and Moderna vaccines in the United States, 90.8% of the AEs were considered non-serious.^45^ A total of 113 deaths were reported to VAERS. However, 65% were among long-term care facility residents (mostly elderly and/or with chronic diseases), and the available information did not suggest any causal relationship between COVID-19 vaccination and death. A case of purpuric rash and thrombocytopenia in a 60-year-old male patient was also reported after vaccination with the Moderna COVID-19 vaccine.^46^ Immune thrombocytopenia has been investigated as a suspected side effect for all approved COVID-19 vaccines.^47^

## DISCUSSION AND RECOMMENDATIONS

In this review, we synthesised the data concerning thromboembolic AEs reported after the roll-out of the COVID-19 vaccines approved in Europe. We have also described how these safety signals led to the total or partial interruption of Vaxzevria and Janssen’s vaccination campaign.

Results indicate that VIPIT is a very rare side effect of Vaxzevria and the Janssen COVID-19 vaccine.^26^,^27^,^48^,^49^ These thrombotic events are potentially severe and fatal. Therefore, this condition must be recognised early by frontline healthcare professionals. Patients with VIPIT may present with CSVT, splanchnic vein thrombosis, or other arterial or venous blood clots. Red flags include persistent and severe headaches, focal neurological symptoms (including new-onset blurred vision), shortness of breath, abdominal or chest pain, swelling and redness in a limb, or pallor and coldness in a limb, occurring within 4–26 days post-vaccination with Vaxzevria or Janssen COVID-19 vaccines.^26^,^33^–^36^ Clinicians presented with a suspected VIPIT case should schedule a haematology consultation to test and start treatment promptly. The true incidence rate of VIPIT is not known. It cannot be directly calculated from pharmacovigilance reports because they lack denominators, the total number of persons or person-times being observed.^50^,^51^

Furthermore, this clinical entity has just been recognised, and it is thus likely to be underdiagnosed and underreported. Thrombotic events are described in different terms in EudraVigilance reports. It would be helpful that doctors report these as a single disease (i.e., VIPIT) in the pharmacovigilance systems. The investigation must proceed, and medicines agencies should consider referring to this as VIPIT in their reports and communication directed to health professionals.

To the best of our knowledge, this is the first study to specifically review the unusual thrombotic events reported in real-world contexts after vaccination against COVID-19 and, additionally, weighed the pros and cons of using these vaccines to make recommendations. It is plausible that the study may have three limitations. Some information was taken from grey literature, including interim reports, news and editorials. As we also included data from spontaneous passive surveillance reports, incidence rates could not be calculated. The inclusion of studies was not exhaustive but covered many major polls and important factors for a comprehensive picture of the phenomenon under investigation.

Nevertheless, unusual thrombotic events combined with thrombocytopenia are considered a very rare side effect of the approved viral vector vaccines; these have been added in the “precautions” section of the product information for Vaxzevria and Janssen COVID-19. Regarding thromboembolic events, mRNA vaccines from Pfizer/BioNTech and Moderna are considered safe and must continue to be used.

Thrombotic cases were mainly observed in young to middle-aged adults, and the reporting rate was not found to be higher in vaccinated older adults than in the non-vaccinated. Moreover, COVID-19 is more likely to be critical and mortal in people over 65 years.^52^ Therefore, considering the risk of SARS-CoV-2 infection and disease complications, VIPIT should not delay vaccine distribution among this priority group, as this is essential to reduce hospitalisations, ICU admissions and deaths due to COVID-19.

Due to the pandemic emergency, EMA has had to grant conditional marketing authorisations to accelerate the authorisation process for the COVID-19 vaccines, which involves rigorous monitoring by the EU pharmacovigilance system. EMA’s safety committee regularly evaluates collected data to confirm that the benefits outweigh the risks of vaccination. This is a dynamic process and, according to the precautionary principle, if safety signals of concern emerge, vaccination campaigns may be temporarily paused. This is not the first time that vaccines are suspended in history. For example, RotaShield, the first vaccine against rotavirus gastroenteritis, was withdrawn after a causal relationship between vaccination and intussusception was established.^53^ Further precautions and contraindications may be defined based on the evidence of risk factors for a severe side effect.

## CONCLUSION

In summary, systematic safety monitoring of COVID-19 vaccines is essential to ensure benefits are superior to the risks. Determinants of VIPIT should be investigated so that evidence-based decisions can be made. Given that vaccination is a preventive measure, safety requirements need to be higher. Safer alternative vaccines should be considered for people with risk factors for VIPIT.
